# Supplementary material for: The Central Fluid Percussion Brain Injury in a Gyrencephalic Pig Brain: Scalable Diffuse Injury and Tissue Viability for Glial Cell Immunolabeling following Long-Term Refrigerated Storage
Source: Biomedicines. 2023 Jun 10;11(6):1682. doi: 10.3390/biomedicines11061682 (PMC10295711; doi:10.3390/biomedicines11061682)
Supplement: Supplementary file 1 [file biomedicines-11-01682-s001.zip › biomedicines-2427459-supplementary.pdf]

Supplemental Table S1: Full Data for individual animals used in this study.

Animal ID	Injury (ATM)	Weight (kg)	ATM/kg	Injury duration (ms)	TAI CC	TAI Thalamus	Microglia Index CC	Microglia Index Thalamus	O2%	PaO2	PaCO2	MABP	pH	Hct	HCO3	Hb
#10	1.64	18.19	0.09		17.46	3.32	4.63	3.88	99.75	617.63	39.00		7.47	29.25	28.73	9.73
#12	2.12	17	0.12	32	76.38	13.95	4.88	4.50	98.50	226.70	36.91	73.05	7.50	32.00	29.20	10.70
#13	1.5	29.5	0.05	33.2	16.13	20.57	4.02	4.38	98.95	461.30	36.85	81.53	7.53	28.00	31.05	9.33
#14	2.13	16.5	0.13	24	359.30	21.90	4.94	4.38	99.90	577.68	36.48	75.20	7.53	32.83	30.97	10.97
#15	1.66	18.4	0.09	28.8	85.63	9.25	3.94	3.88	99.90	605.74	37.73	76.00	7.54	24.29	32.54	8.14
#17	1.69	14.9	0.11	32.8	6.21	7.33	4.81	2.50	99.02	342.90	37.60	72.11	7.51	27.00	30.45	8.98
#18	1.83	17.5	0.10		80.96	14.53	4.31	4.75	99.66	494.06	40.03	85.95	7.50	27.29	31.54	9.07
#19	1.53	19.2	0.08	31.6	9.92	5.92	3.31	2.75	99.90	561.27	37.86	64.66	7.54	30.43	32.40	10.19
#20	1.39	24.1	0.06	33.2	29.33	6.98	3.75	4.63	99.90	600.45	38.25	69.51	7.53	27.33	32.07	9.10
#21	1.61	21.2	0.08	30	75.08	17.43	4.56	4.50	99.90	588.96	36.86	86.37	7.53	29.80	31.30	9.92
#22	1.66	19.3	0.09	32	26.25	9.33	3.44	4.50	99.90	596.66	37.37	74.13	7.52	25.57	30.53	8.53
#23	1.77	19.3	0.09	26	35.58	4.42	3.88	3.38	99.90	560.72	37.73	89.32	7.52	29.33	31.10	9.80
#24	1.55	24.4	0.06	32.2	5.91	4.47	3.75	2.88	99.34	329.16	37.20	74.70	7.53	33.80	31.30	11.30
#26	1.82	19.6	0.09	34.6	3.29	6.50	3.44	0.75	98.23	362.11	37.83	93.44	7.49	22.57	29.30	7.51
#27	1.67	20.4	0.08	31.8	22.17	13.72	3.69	4.25	98.60	388.98	38.70	83.88	7.51	26.38	31.41	8.81
#28	1.61	19.8	0.08	32.4	26.13	7.93	3.63	3.63	99.90	518.01	36.63	87.49	7.50	18.00	28.32	6.06
#30	1.61	21.4	0.08	31.6	14.13	7.28	3.71	3.50	99.90	479.95	38.73	90.60	7.50	31.71	30.24	10.57
#31	1.58	21.4	0.07	23.2	26.13	10.75	2.63	4.13	99.90	546.95	35.15	80.44	7.54	24.63	30.00	8.19
#16	0	15.3	0.00	0	1.33	1.57	0.94	1.88	98.81	161.83	36.36	70.75	7.52	23.14	29.47	7.71
#25	0	17.7	0.00	0	0.50	0.98	1.19	0.75	98.86	381.93	38.89	109.01	7.46	31.86	28.29	10.59
#29	0	24.4	0	0	0.09	3.00	1.38	0.88	99.90	535.46	38.53	78.65	7.48	28.63	28.61	9.53

ATM=atmospheric pressure, ms=milliseconds, TAI=traumatic axonal injury, CC=corpus callosum, MABP=mean arterial blood pressure. All blood gas and MABP readouts from the last hour prior to sacrifice (5-6hr post-injury).
